# A Novel Variant in *VPS13B* Underlying Cohen Syndrome

**DOI:** 10.1155/2023/9993801

**Published:** 2023-04-12

**Authors:** Abrar Hussain, Anushree Acharya, Thashi Bharadwaj, University Of Washington Center For Mendelian Genomics, Suzanne M. Leal, Abdul Khaliq, Asif Mir, Isabelle Schrauwen

**Affiliations:** ^1^Human Molecular Genetics Lab, Department of Biological Science, Faculty of Basic and Applied Sciences, International Islamic University, Islamabad 44000, Pakistan; ^2^Center for Statistical Genetics, Gertrude H. Sergievsky Center, Department of Neurology, Columbia University Medical Center, New York 10032, USA; ^3^Department of Genome Sciences, University of Washington, Seattle, WA 98195, USA; ^4^Taub Institute for Alzheimer's Disease and the Aging Brain, and the Department of Neurology, Columbia University Medical Center, New York, 10032 NY, USA

## Abstract

Pathogenic variants in vacuolar protein sorting 13 homolog B (*VPS13B*) cause Cohen syndrome (CS), a clinically diverse neurodevelopmental disorder. We used whole exome and Sanger sequencing to identify disease-causing variants in a Pakistani family with intellectual disability, microcephaly, facial dysmorphism, neutropenia, truncal obesity, speech delay, motor delay, and insomnia. We identified a novel homozygous nonsense variant c.8841G > A: p.(W2947^∗^) in *VPS13B* (NM_017890.5) which segregated with the disease. Sleep disturbances are commonly seen in neurodevelopmental disorders and can exacerbate medical issues if left untreated. We demonstrate that individuals with Cohen syndrome may also be affected by sleep disturbances. In conclusion, we expand the genetic and phenotypic features of Cohen syndrome in the Pakistani population.

## 1. Introduction

Cohen syndrome (CS) is a rare heritable autosomal recessive disorder that includes intellectual disability (ID), developmental delay, microcephaly, and hypotonia. Myopia and retinal dystrophy are additional common characteristics, but hypermobility, facial dysmorphism, and a bulbous nasal tip are rarer features [1]. Cohen syndrome has a wide range of clinical characteristics among those who are affected. Additional signs and symptoms in certain people with this syndrome include neutropenia, autistic-like features, and truncal obesity. Affected individuals may also have small hands, feet, and fingers [2]. CS is diagnosed at a frequency of 0.7% in individuals with unexplained intellectual disability [3].

CS is caused by variants in *VPS13B* (also known as COH1), which codes for vacuolar protein sorting 13 homolog B. It is a 4022-amino-acid transmembrane protein that is located on chromosome 8 (8q22.2). The protein encoded by this gene is a Golgi-associated peripheral membrane protein involved in Golgi integrity and homeostasis, as well as membrane transport and it is a member of the VPS13 protein family, which is extremely well preserved in eukaryotic cells [4]. Chorea acanthocytosis (OMIM: 200150), rapidly progressive, early-onset autosomal recessive Parkinson's disease (OMIM: 616840), and spinocerebellar ataxia, autosomal recessive 4 (OMIM: 607317) are all possible outcomes of loss of function variants in other VPS13 family members [5].

With the fast advancement of high-throughput sequencing technology, exome sequencing has enabled patients with subtle clinical symptoms to get an early and precise molecular diagnosis, thereby enhancing patient quality of life and simplifying genetic counseling [6]. Due to its massive benefits such as high efficiency, cost, and high accuracy, exome sequencing has been widely used in clinical practice and research [7]. In this study, we investigated a large consanguineous pedigree with ID from Pakistan via exome sequencing.

## 2. Methods

This research was performed in accordance with the Declaration of Helsinki. Ethical approval for the study was obtained from the Institutional Review Board (IRB) of the involved institutions, the International Islamic University Islamabad (IIUI/BIBT/FBAS-2022/77) and Columbia University (IRB-AAAS3433). Informed consent was obtained from healthy adult subjects, the parents/legal guardians of minor subjects, and the ID patients in this study to publish the findings of the study.

### 2.1. Clinical Assessment

**Figure 1:**
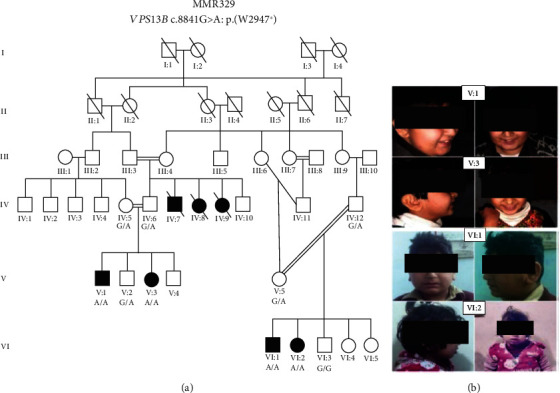
(a) The autosomal recessive consanguineous pedigree studied here and the segregation of the *VPS13B* [c.8841G > A: p.(W2947^∗^)] variant. Squares symbolize the male individuals, circles denote female individuals, and filled square and circle indicates the affected individuals. Double lines denote a consanguineous marriage, and the crossed line specifies the deceased individual. (b) Facial images of the affected individuals. All individuals shared facial dysmorphism features such as a bulbous nasal tip, a prominent nose root, a short philtrum, narrow (mouth) roof (palate), prominent upper incisors, large ears, thick eyebrows, long thick eyelashes, and wave-shaped eyelids. Eye misalignment is seen in V : 1 and VI : 2 individuals only.

A large consanguineous family with ID (MMR-329) of Pashtun ancestry was ascertained in Pakistan (Figure 1(a)). Pedigree information was recorded up to six generations, with a total of seven affected individuals of whom three (IV : 7, IV : 8, and IV : 9) are deceased. Living affected individuals were examined by a local neurologist and psychiatrist (Figure 1(b)). The phenotypic information of all affected members of the family was noted in detail (Table 1) which included age, sex, height, ID, facial dysmorphism, developmental delay, and psychomotor delay features.

### 2.2. Exome Sequencing

The extraction of genomic DNA was done from the blood of the patients and unaffected members of the family by standard organic methods of phenol-chloroform for genetic analysis. DNA samples from two patients (V : 3 and VI : 1) were exome sequenced using the Twist+RefSeq library preparation kit (Twist Bioscience, San Francisco, CA, USA). Barcoded libraries were pooled, and sequencing was performed on the NovaSeq 6000 (Illumina Inc., San Diego, CA, USA) with an average on-target coverage of 40X.

### 2.3. Bioinformatic Analysis

Reads were aligned to GRCh37/hg19 using the Burrows-Wheeler aligner (BWA) [8]. Duplicate removal, indel-realignment, quality recalibration, and variant detection and calling were performed using Picard and the genome analysis toolkit (GATK) following the GATK best practices workflows [9]. Variants were annotated using ANNOVAR [10]. The criteria for variant selection included a minor allele frequency (MAF) of <0.005 in each population of gnomAD [11], a CADD-Phred score of >20 for missense variants, exonic variants, splice sites (±12 bp), and fitting an inheritance model consistent with the pedigree (autosomal recessive) [12]. Bioinformatic prediction tools were used to evaluate the effect of the variant on the protein (Table 2). These tools included MutationTaster [13], FATHMM-MKL [14], DANN [15], LRT-Pred [16], CADD [17], GERP [18], and VEST4 [19].

### 2.4. Sanger Sequencing

The *VPS13B* variant from the WES data was validated by Sanger sequencing and tested in additional family members to confirm its segregation with the disease. Primers were designed through Primer3 (https://bioinfo.ut.ee/primer3-0.4.0/). PCR-amplified products were purified by ExoSAP-IT (USB Corp., Cleveland, OH, USA) and were sequenced using the BigDye Terminator v3.1 Cycle Sequencing Kit followed by capillary electrophoresis on an ABI 3730 DNA Analyzer (Applied Biosystems Inc., Foster City, CA USA). The DNA sequences were then aligned to the reference genome sequence using the CodonCode Aligner v7.1.2 (CodonCode Corp., Centerville, MA, USA).

## 3. Results

### 3.1. Clinical Findings

A description of the features seen in each affected individual (V : 1, V : 3, VI : 1, and VI : 2) is listed in detail in Table 1. Briefly, these individuals presented with ID and other clinical features including microcephaly, neutropenia, facial dysmorphism, developmental delay, psychomotor delay, a bulbous nasal tip, truncal obesity, small hands, feet, and fingers. One patient (VI : 2) of this CS family showed additional features including hypotonia, muscle atrophy, and insomnia.

### 3.2. Exome Sequencing and Follow-Up

**Figure 2:**
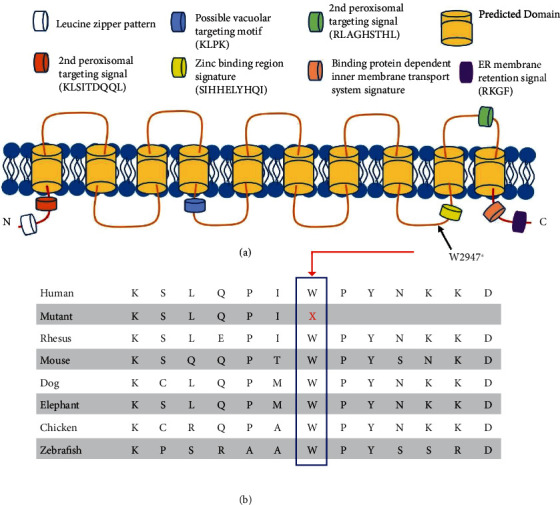
(a) Prediction of membrane topology and domains of VPS13B. The identified variant in the CS patients is shown by a black arrow. (b) The human VPS13B protein sequence (2941-2953) aligned with orthologous protein sequences of different species indicating evolutionary conservation of the C-terminal domain of VPS13B.

Exome sequencing of two affected individuals (V : 3 and VI : 1) in the family revealed a novel homozygous nonsense variant c.8841G > A (NM_017890.5) located in exon 48 out of 62 in the *VPS13B* gene (Supplementary Figure 1b) at position 8q22.2 (Supplementary Figure 1a). The variant altered the tryptophan amino acid into a premature stop codon p.(W2947^∗^). The average on-target coverage was 40x, and 24x and 33x at the *VPS13B* c.8841G > A site for both exomed individuals. Sanger sequencing verified the segregation of this variant in the pedigree (Supplementary Figure 1c). This novel nonsense mutation is absent in gnomAD [20], 1000 genomes [21], GME Variome [22], Kaviar [23], ABraOM [24], AllofUs [25], and TOPMed [26], and bioinformatic tools predict that it significantly damages the function of the VPS13B protein (Table 2). A protein alignment of amino acid sequence 2941 to 2953 of the wild-type human and mutant VPS13B with orthologous proteins of the different species indicated an evolutionary conserved of the C-terminal domain (Figure 2(b)), and the GERP++RS score was 5.98 (Table 2). The variant was classified as pathogenic based on standard guidelines of the American College of Medical Genetics (ACMG) [27].

## 4. Discussion

CS is a rare autosomal recessive disorder mainly characterized by ID, impaired growth, microcephaly, neutropenia, truncal obesity, and facial dysmorphism, with other additional features including myopia and retinal dystrophy, small fingers, and a bulbous nasal tip [28]. In this study, we observed phenotypic diversity among the affected individuals of the same family which includes motor delay (VI : 1 and VI : 2), strabismus (V : 1 and VI : 2), and hypotonia, muscle atrophy, and insomnia (VI : 2). Furthermore, all affected individuals manifested characteristic CS features including moderate ID, microcephaly, neutropenia, developmental delay, truncal obesity, a bulbous nasal tip, small fingers, and facial dysmorphism. Previous studies in Pakistani consanguineous families underlying CS have described additional features associated with Cohen syndrome including cerebellar hypoplasia [29], autistic-like features [30], and individuals with a milder form of CS [31]. Moreover, in the present study, we found a previously undescribed feature (insomnia) in CS in the affected individual (VI : 2). This individual has difficulty falling asleep, repeated awakenings with difficulty returning to sleep, or sleep that is nonrestorative or poor in quality. Sleep disturbances such as insomnia are a common feature in many neurodevelopmental disorders (NDDs) [32] such as the Angelman syndrome (AS), autism spectrum disorder (ASD), the Smith-Magenis syndrome (SMS), the Prader-Willi syndrome (PWS), tuberous sclerosis complex (TSC) [33], Fragile X syndrome (FXS), the Williams syndrome (WS), and the Rett syndrome (RTT). Not only are sleep disturbances significantly higher in NDDs than in age-matched unaffected children but they also often last longer as well, such as into adolescence and adulthood [32]. Untreated sleep disorders can aggravate their medical issues, and early interventions may be beneficial to the patient's overall health.

In this study, we report a novel genetic variant in *VPS13B* [NM_017890.5, c.8841G > A: p.(W2947^∗^)] in an autosomal recessive consanguineous Pakistani CS family. Over 200 variants have been reported worldwide in multiple domains of *VPS13B* associated with CS [34]. *VPS13B* protein has ten transmembrane domains and a potential vacuolar targeting motif, an endoplasmic reticulum retention signal on the C-terminus and two peroxisomal matrix protein targeting signal 2 (PTS2) consensus sequence both on the N- and C-terminus (Figure 2(a)) [35]. Our nonsense variant resides in between the 8th and 9th transmembrane domains of *VPS13B*, and at this position, the variant is likely targeted via nonsense mRNA-mediated decay (NMD), resulting in no or limited truncated protein expression.

In conclusion, we report a novel nonsense variant in *VPS13B* associated with CS in a large Pakistani family which displayed phenotypic variability and an expanded phenotype. This study will help facilitate the diagnosis and genetic counseling of families with CS-related features in the Pakistani population.

## Figures and Tables

**Table 1:** Clinical details of the affected individuals.

Patient features	V : 1	V : 3	VI : 1	VI : 2
Sex	Male	Female	Male	Female
Consanguinity of parents	First cousin		First cousin	
Age (years)	13	7	14	5
Height (cm)	150	117	136	88
Age at first words (months)	20	19	20	19
Age at walking (months)	32	28	33	28
Intellectual disability	Moderate	Moderate	Moderate	Moderate
Microcephaly	+	+	+	+
Neutropenia	+	+	+	+
Hypotonia	-	-	-	+
Motor delay	-	-	+	+
Speech delay	+	+	+	+
Muscular atrophy	-	-	-	+
Insomnia/sleep disturbances	-	-	-	+
Neonatal feeding difficulties	+	+	+	+
Strabismus	+	-	-	+
Wave shape eyelid	+	+	+	+
Long and thick eyelashes	+	+	+	+
Large ear	+	+	+	+
Prominent nose root	+	+	+	+
Prominent upper central incisors	+	+	+	+
Narrow roof mouth	+	+	+	+
Short philtrum	+	+	+	+
Truncal obesity	+	+	+	+
Small hands, feet, and fingers	-	+	-	+

**+** Present, - absent.

**Table 2:** Bioinformatic prediction results for *VPS13B* c.8841G > A: p.(W2947^∗^).

Bioinformatic tool	Prediction	Score	Prediction range
MutationTaster	Disease causing	1	0-1 (close to 1 indicating disease causing)
FATHM-MKL	Damaging	0.998	0-1 (close to 1 indicating damaging)
DANN	Damaging	0.996	0-1 (close to 1 indicating damaging)
LRT-Pred	Deleterious	0.860	0-1 (close to 1 indicating deleterious)
CADD	Deleterious	45	1-99 (>20 deleterious)
GERP	Deleterious	5.98	>4 highly conserved (deleterious)
VEST4	Mutated	0.861	0-1 (close to 1 indicating the functional mutation

## Data Availability

The datasets used and/or analysed during the current study are available from the corresponding authors on reasonable request. The variant identified in this study has been deposited into ClinVar (SCV002564668). Bio workflows used are available at https://github.com/cumc/bioworkflows.

## Abbreviations

*VPS13B*: Vacuolar protein sorting 13 homolog B
CS: Cohen syndrome
WES: Whole exome sequencing
ID: Intellectual disability
COH1: Cohen syndrome 1
OMIM: Online Mendelian Inheritance in Man
NGS: Next generation sequencing
DNA: Deoxyribonucleic acid
BWA: Burrows-Wheeler aligner
GATK: Genome analysis tool kit
ANNOVAR: Annotate variation
MAF: Minor allele frequency
CADD: Combined annotation-dependent depletion
DANN: Deleterious annotation of genetic variants using neural networks
GERP: Genomic evolutionary rate profiling
VEST: Variant effect scoring tool
PCR: Polymerase chain reaction
gnomAD: Genome aggregation database
GME: Greater Middle East
Kaviar: Known variants
ABraOM: Brazilian genomic variants
TOPMed: Trans-Omics for Precision Medicine
AS: Angelman syndrome
ASD: Autism spectrum disorder
SMS: Smith-Magenis syndrome
PWS: Prader-Willi syndrome
TSC: Tuberous sclerosis complex
FXS: Fragile X syndrome
WS: Willian syndrome
RTT: Rett syndrome
C: Carbon
N: Nitrogen
PTS2: Protein targeting signal 2
NMD: Nonsense mediated decay
ACMG: American College of Medical Genetics.

## Supplementary Materials

Supplementary Materials Figure 1: a. Cytogenetic location of the *VPS13B* gene. b. Schematic representation of *VPS13B* in which the light grey color shows intronic regions while dark grey indicates the exons. Indicated in red is mutated exon 48. c. The Sanger chromatograms of *VPS13B* c.8841G > A in healthy carriers (V : 2, IV : 5, V : 5, and IV : 6 and IV : 12), homozygous wild type (VI : 3), and affected individuals (V : 1, V : 3, VI : 1, and VI : 2) in a homozygous state.
