# Supplementary material for: A Novel Variant in VPS13B Underlying Cohen Syndrome
Source: Biomed Res Int. 2023 Apr 12;2023:9993801. doi: 10.1155/2023/9993801 (PMC10115529; doi:10.1155/2023/9993801)
Supplement: Supplementary Materials — Figure 1: a. Cytogenetic location of the VPS13B gene. b. Schematic representation of VPS13B in which the light grey color shows intronic regions while dark grey indicates the exons. Indicated in red is mutated exon 48. c. The Sanger chromatograms of VPS13B c.8841G > A in healthy carriers (V : 2, IV : 5, V : 5, and IV : 6 and IV : 12), homozygous wild type (VI : 3), and affected individuals (V : 1, V : 3, VI : 1, and VI : 2) in a homozygous state. [file 9993801.f1.docx]

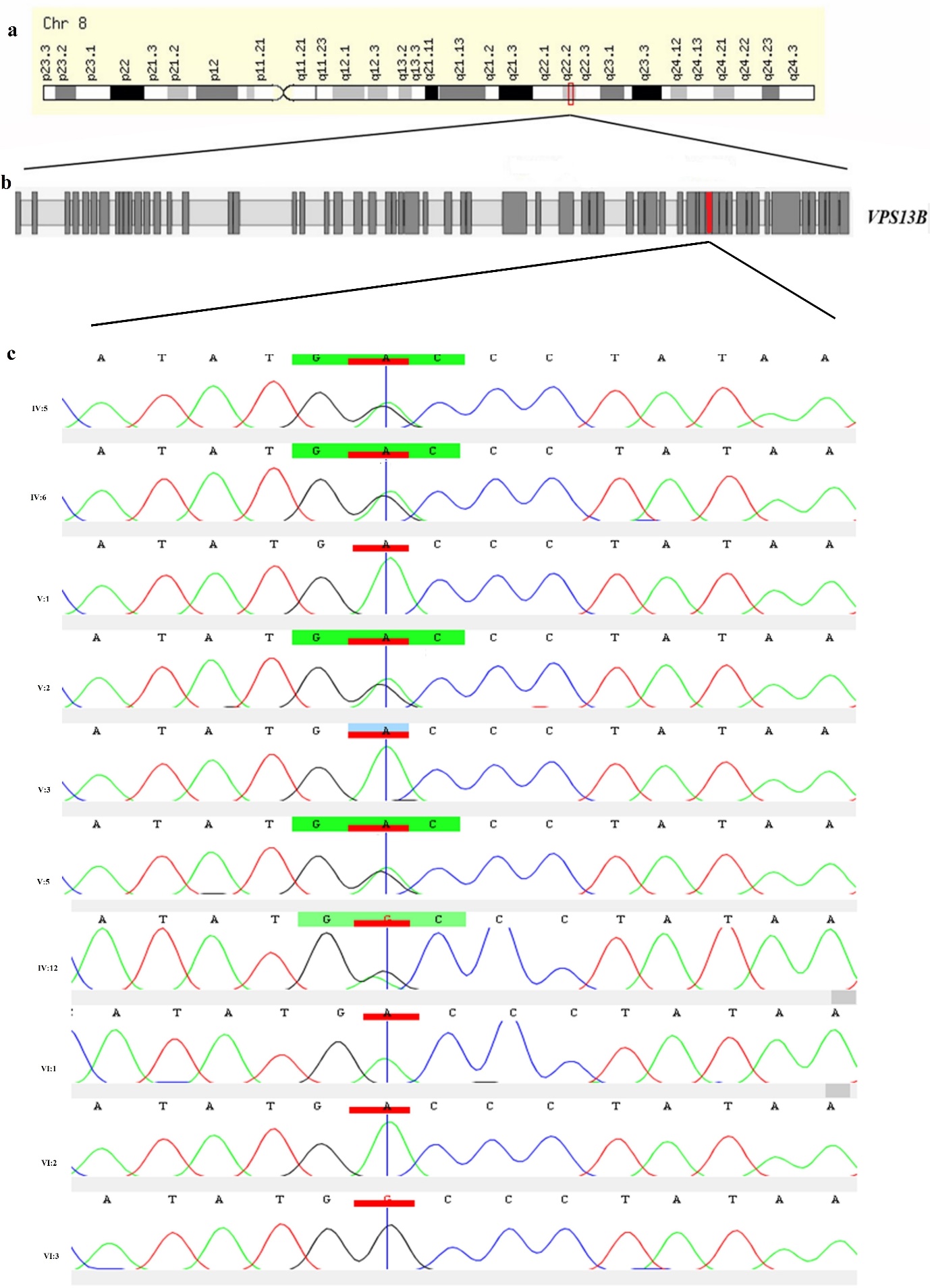


**Supplementary Figure 1: a.** Cytogenetic location of the *VPS13B* gene; **b.** Schematic representation of *VPS13B* in which the light grey color shows intronic regions while dark grey indicates the exons. Indicated in red is mutated exon 48; **c.** Sanger chromatograms of *VPS13B* c.8841G>A in healthy carriers (V:2, IV:5, V:5 & IV:6 & IV:12), homozygous wild type (VI:3), and affected individuals (V:1, V:3, VI:1 & VI:2) in a homozygous state.
